# Development of the Engage with Impact Toolkit: A comprehensive resource to support the evaluation of patient, family and caregiver engagement in health systems

**DOI:** 10.1111/hex.13742

**Published:** 2023-03-21

**Authors:** Julia Abelson, Laura Tripp, Maggie MacNeil, Amy Lang, Carol Fancott, Rebecca Ganann, Marisa Granieri, Cathie Hofstetter, Bernice King, Betty‐Lou Kristy, Alies Maybee, Maureen Smith, Jeonghwa You, The Evaluating Patient Engagement Working Group

**Affiliations:** ^1^ Public and Patient Engagement Collaborative, Department of Health Research Methods, Evidence and Impact McMaster University Hamilton Ontario Canada; ^2^ School of Nursing McMaster University Hamilton Ontario Canada; ^3^ Patient Oriented Research Canadian Institutes of Health Research, Government of Canada Ottawa Ontario Canada; ^4^ Patient Engagement & Partnerships Healthcare Excellence Canada Ottawa Ontario Canada; ^5^ Independent Patient Partner Ontario Canada; ^6^ Minister's Patient and Family Advisory Council, Ministry of Health, Government of Ontario Toronto Ontario Canada

**Keywords:** evaluation tools, family and caregiver engagement, impact evaluation, patient, public and patient engagement

## Abstract

**Introduction:**

Recent shifts in the patient, family and caregiver engagement field have focused greater attention on measurement and evaluation, including the impacts of engagement efforts. Current evaluation tools offer limited support to organizations seeking to reorient their efforts in this way. We addressed this gap through the development of an impact measurement framework and accompanying evaluation toolkit—the Engage with Impact Toolkit.

**Methods:**

The measurement framework and toolkit were co‐designed with the Evaluating Patient Engagement Working Group, a multidisciplinary group of patient, family and caregiver partners, engagement specialists, researchers and government personnel. Project activities occurred over four phases: (1) project scoping and literature review; (2) modified concept mapping; (3) working group deliberations and (4) toolkit web design.

**Results:**

The project scope was to develop a measurement framework and an evaluation toolkit for patient engagement in health systems that were practical, accessible, menu‐driven and aligned with current system priorities. Concept mapping yielded 237 impact statements that were sorted, discussed and combined into 81 unique items. A shorter list of 50 items (rated 8.0 or higher out of 10) was further consolidated to generate a final list of 35 items mapped across 8 conceptual domains of impact: (1) knowledge and skills; (2) confidence and trust; (3) equity and inclusivity; (4) priorities and decisions; (5) effectiveness and efficiency; (6) patient‐centredness; (7) culture change and (8) patient outcomes and experience. Working Group members rated the final list for importance (1–5) and identified a core set of 33 items (one for each of the 8 domains and 25 supplementary items). Two domains (priorities and decisions; and culture change) yielded the highest overall importance ratings (4.8). A web‐based toolkit (www.evaluateengagement.ca) hosts the measurement framework and related evaluation supports.

**Conclusion:**

The Engage with Impact Toolkit builds on existing engagement evaluation tools but brings a more explicit focus to supporting organizations to assess the impacts of their engagement work.

**Patient Contribution:**

Patient, family and caregiver partners led the early conceptualization of this work and were involved at all stages and in all aspects of the work. As end‐users of the toolkit, their perspectives, knowledge and opinions were critical.

## 1 INTRODUCTION

Recent advances in the field of patient, family and caregiver engagement in research and health systems have focused greater attention on its measurement and evaluation.^1^, ^2^ As Boivin et al. state in their BMJ editorial, ʻif we are serious about involvement, we need to be equally serious about evaluation’.^1^ Just as the goals for engaging patients vary, so too do the goals for its evaluation. Early interest in evaluation has primarily focused on improving the quality of engagement practices. As engagement practices have become more firmly embedded in organizations, the goals for evaluation have shifted to assessing the outcomes and impacts of these activities and processes.^3^, ^4^

A growing number of tools for evaluating patient engagement have been developed over the last decade, followed by several systematic reviews that have attempted to summarize them and assess various aspects of their quality. Reviewing this literature is challenging because of the multiplicity of review objectives and the diversity of evaluation resources that range from conceptual frameworks and measures to practical supports that include data collection instruments. Boivin et al. systematically reviewed and appraised 27 tools used to evaluate the patient and public engagement in research and health system decision‐making.^5^ Most tools were designed to support the quality improvement of engagement activities within organizations and health research teams. Deficiencies were noted across the tools in terms of lack of scientific rigour (only 11% were explicitly informed by a literature review and just 7% were tested for reliability) and the absence of public and patient engagement in their design and reporting (56% of the time but mainly in the piloting stage, and only 18.5% of tools were designed to report evaluation results to patients and the public).^5^ If patient and public engagement is founded on the principles of reciprocity and equal partnership, its evaluation should uphold the same principles.

Dukhanin et al.^6^ focused at a more microlevel by developing a taxonomy of 116 process and outcome metrics for evaluating engagement in patient, public, consumer and community engagement in organization‐, community‐ and system‐level healthcare decision‐making. Their taxonomy was then used to assess the coverage of evaluation metrics in 23 evaluation tools published between 1973 and 2015. Tools varied in their coverage of the taxonomy; nearly twice as many process metrics were identified (72) compared to outcome metrics (44). Among the outcome metrics, more addressed internal outcomes (the effect of engagement on the organization itself, its services or engagement participants) as compared to external outcomes (i.e., the effect of engagement beyond the organization where it occurs). Of particular interest is the authors' proposal for a maturity model approach to evaluation that includes three levels that organizations can progress through over time.^6^ Dukhanin et al.^6^ note important deficiencies in the tools and identified priorities for future research, namely, (i) the lack of rigorous testing of the tools supporting the need to better understand the validity and reliability of the tools and metrics in different healthcare contexts and (ii) the diversity of metrics identified, suggesting the need for consensus‐building to identify and disseminate core metrics.

Greenhalgh et al.^7^ review of 65 frameworks for supporting, evaluating and reporting patient and public engagement in health research revealed the diverse and theoretically heterogeneous nature of engagement literature and concluded that the diversity in purposes and scientific underpinnings of existing frameworks limited transferability across contexts. They concluded that ʻa single, one‐size‐fits‐all framework may be less useful than a range of resources that can be adapted and combined in a locally generated co‐design activity’ developed with patient collaborators.^7^

Current engagement evaluation tools offer limited support to organizations seeking to build incrementally towards impact measurement. Most tools with an exclusive focus on impact measurement have been developed for application to health research. The lack of engagement evaluation tools designed specifically for patient engagement in health system planning, design and decision‐making is a notable gap. We address these gaps through the development of the Engage with Impact Toolkit, an online evaluation toolkit including an impact measurement framework and evaluation supports designed by and for a broad range of health system partners including engagement leads; patient, family and caregiver partners and evaluation specialists working at different levels of health system planning.

### 1.1 Setting and context

This research was conducted in Ontario, Canada. In 2019, the provincial government introduced a major health system transformation designed to foster more integrated care through the establishment of Ontario Health Teams (OHTs). Comparable to the accountable care organizations in the United States, OHTs are ʻgroups of providers and organizations that are clinically and fiscally accountable for delivering a full and coordinated continuum of care to a defined geographic population’.^8^ OHTs are expected to ʻuphold the principles of patient partnership, community engagement and system co‐design… (and) meaningfully engage and partner with – and be driven by the needs of – patients, families, caregivers and the communities they serve’.^8^ Early experience with OHTs suggests that patient, family and caregiver partners are engaging and partnering within these new systems in different ways and with varied success. The need for frameworks and metrics to link these activities to relevant outcomes and impacts has also been documented,^9^, ^10^ reinforcing similar messages from the broader engagement evaluation literature.

The Public and Patient Engagement Collaborative at McMaster University is a supporting partner for the implementation of OHTs and provides engagement evaluation support across two of the eight building blocks of the OHT model: (i) patient partnership and community engagement and (ii) performance measurement, quality improvement and continuous learning. The Public and Patient Engagement Collaborative's work with OHTs across these three building blocks laid the foundation for the development of the Engage with Impact Toolkit (henceforth, the Toolkit). In this context, patient engagement refers to patients, families and caregivers collaborating with members of the health system (e.g., healthcare professionals, health system administrators, government employees and others) to improve healthcare quality.^11^

## 2 METHODS

### 2.1 Study design and participants

At the request of the Ministry of Health, the Public and Patient Engagement Collaborative was tasked with leading a Working Group of patient, family and caregiver partners, engagement specialists, researchers and government personnel to develop a measurement framework for evaluating patient engagement in the new OHT context. Working Group members were recruited from a list of 41 health system partners who attended a patient engagement evaluation workshop in April 2019, co‐led by the Ministry of Health and the Chair of the Minister's Patient and Family Advisory Council. New members were added to include representation from the newly established OHTs. Collectively, the 27‐member Working Group held a broad range of engagement and evaluation experience and expertise both within and outside of the OHT context. The Working Group broadened the original mandate it was given to focus on the development of a patient engagement evaluation toolkit that would meet the needs of a broad range of health system partners. Working Group members were involved in all stages of the project from the early scoping phases where the aims and objectives for the evaluation framework and toolkit were jointly determined through to the design and release of the final toolkit. Patient, family and caregiver partners were offered an honorarium of $125 per meeting as compensation for their contributions. Other members of the working group were not offered honoraria as they participated as part of their employment with their respective organizations.

**Figure 1:**
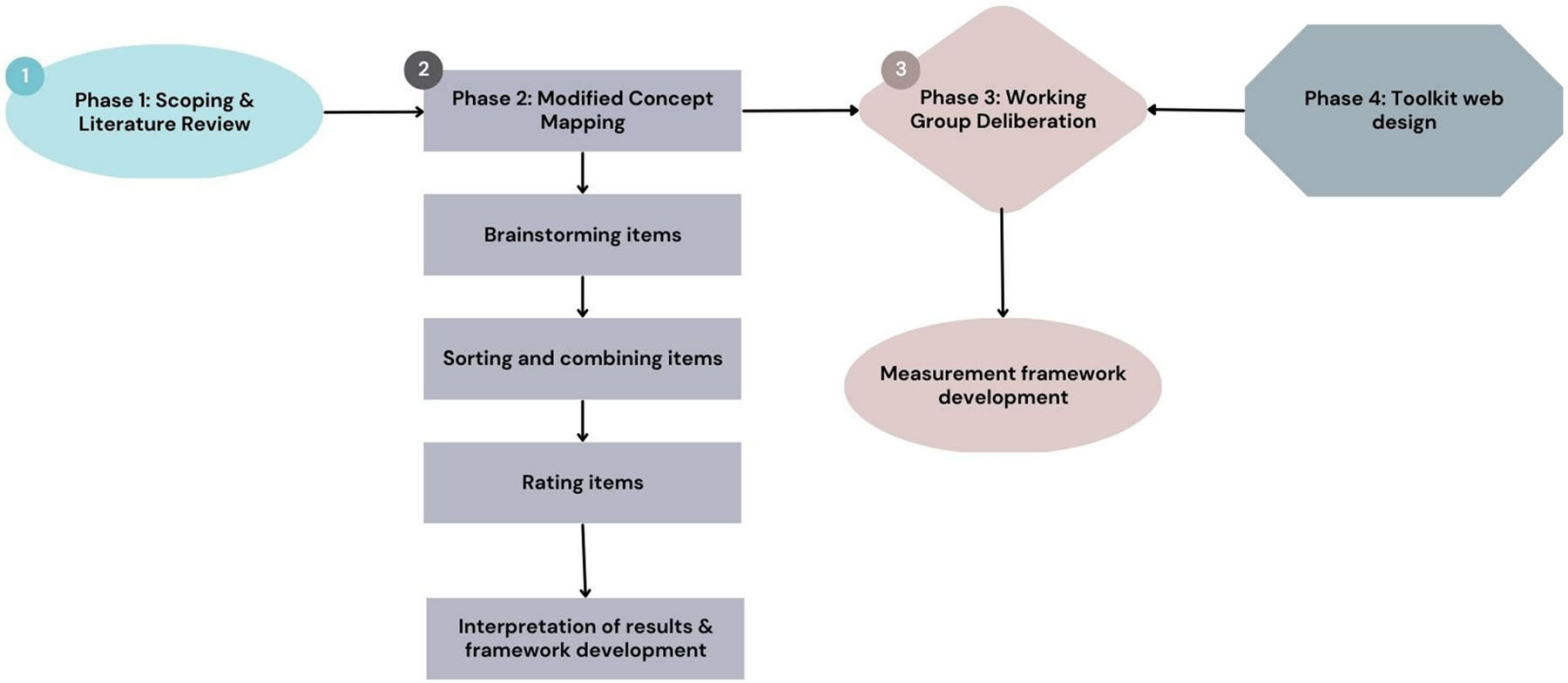
Toolkit development process.

The framework was developed through three phases: (1) project scoping and literature review; (2) modified concept mapping and (3) working group deliberations. A separate phase involved the development of the online toolkit to house the framework (see Figure 1). This work was reviewed and approved by the Hamilton Integrated Research Ethics Board Project # 8360.

### 2.2 Phase 1: Project scoping and literature review

#### 2.2.1 Project scoping

The Working Group established the scope and scale of the project as well as the following guiding principles for the final product: the toolkit would be (1) practical (not overly technical or academic); (2) accessible and easy to use, so it would appeal to a broad range of users; (3) menu‐driven, allowing users and organizations to determine which part of the toolkit would be most useful to them, given their stage of development and maturity in their engagement work and (4) would align with other relevant initiatives and measurement requirements for the OHTs, including advancing the quadruple aim^12^ and funding‐related performance measurement and engagement requirements. Research team members brought methodological rigour to the work to support the development of a valid and robust tool.

#### 2.2.2 Literature review

The research team conducted a rapid literature review focused on synthesized evidence to identify impacts of patient, family and caregiver engagement developed and applied across the health research to knowledge translation and implementation pipeline. A targeted search of the English‐language peer‐reviewed literature and grey literature was carried out.^13^, ^14^ Search parameters included a focus on review articles and frameworks, published in 2019 or earlier using the following search terms: ‘public OR citizen OR patient OR family OR caregiver’ AND ‘engagement OR involvement’ AND ‘evaluation OR assessment’ AND ‘impact’. Results were shared with the Working Group and used to identify a preliminary list of patient engagement impacts. The research team iteratively scanned the literature over the course of the project to identify additional potentially relevant contributions.

### 2.3 Phase 2: Modified concept mapping process

The literature review was augmented by a modified concept mapping process. Concept mapping is a participatory research method used to engage key partners in brainstorming, idea sorting and interpretation of results^15^ and has been used successfully in the Ontario context to integrate patient perspectives to inform health system decision‐making.^16^ We adapted this method to the Working Group's structure, mandate and timeframe (the details of each stage are described below).

#### 2.3.1 Brainstorming items

Working Group members, in addition to a larger sample of patient engagement community members reached through snowball sampling, were invited to participate in the brainstorming stage. Working Group members identified the need to expand the size and diversity of the brainstorming group to ensure that a larger and more diverse set of perspectives and experiences would be captured. Each Working Group member was invited to identify up to five patient partners, engagement leads, health system leads or others working in the field in Ontario to be invited to participate in the brainstorming stage. Once the list of brainstorming participants was finalized, links to the online brainstorming survey were emailed to participants by a member of the research team. A focus prompt is used to structure the brainstorming stage. The focus prompt was a survey question that asked respondents to complete the following sentence: ʻWhen patient, family and caregiver partners are engaged in health system organizations, the impact that patient engagement and partnering has on … is…’. The sentence was asked for each of the following levels of impact: people, programmes, organizations and health systems. Respondents were asked to identify as many impacts as they could for each statement.

#### 2.3.2 Sorting and combining items

Research team members sorted and combined the impact statements (henceforth, items) (e.g., duplicates removed, similar items consolidated). These items, along with those identified through the literature in phase 1, were categorized by four levels of impact: people (individuals involved in an engagement activity—patient partners, staff, etc.), programme (programme design and delivery, governance decisions), organization (the health system organization—e.g., the OHT, hospital, etc.) and health system (e.g., the provincial health system)—an organizing framework commonly used in the programme evaluation literature that was adapted to the health systems context.^17^

#### 2.3.3 Rating items

A second survey was administered to the same group of respondents to rate the statements in answer to the following question: *How important is it to measure this impact?* Respondents rated the importance of measuring each item from 1 to 10 (allowing for decimal values), where 1 was least important and 10 was most important. Respondents were reminded that the focus was on the importance of measuring the impact, not the importance of the impact itself. Statements were presented within their level groups (i.e., people, programmes, organizations and health systems). Additionally, respondents were asked to identify any additional impacts that were not presented but should have been, in each category. Average ratings for each item were calculated and used to identify the impact domains and items to be considered for the measurement framework.

#### 2.3.4 Interpretation of results and framework development

Following concept mapping methodology, where research team members and concept mapping contributors interpret ideas together in a facilitated group meeting,^15^ results of the brainstorming, sorting and rating processes were shared, discussed and interpreted through a virtual working group meeting and several one‐on‐one discussions with Working Group members virtually and by e‐mail. These discussions were used to identify key conceptual categories and further consolidate items as part of the measurement framework development process described in further detail below.

### 2.4 Phase 3: Working group deliberations and measurement framework development

Concept mapping results were presented to the Working Group for discussion and deliberation. The research team presented a preliminary organizing framework of impact domains and items that were further refined. Items that were rated 8.0 or higher (out of 10) in terms of their importance to be measured were chosen for inclusion in a final survey to refine the list of items in each domain.

A final survey of Working Group members invited them to rate each item (within each domain) on a scale of 1–5, where 1 indicated that the item was not important at all (and could be removed from the framework) and 5 indicated that the item is extremely important and should be included as a core item within the framework.

### 2.5 Phase 4: Toolkit web design

The concept mapping results were integrated with the feedback from the working group deliberations to inform the development of the web‐based toolkit (the Engage with Impact Toolkit). The structure and overall design concept for the toolkit were determined by the Working Group. The content was drafted by the research team, including the development of a database of survey and interview questions and document review approaches for each of the identified items. Working Group members had opportunities to review the prototype website independently and during a virtual meeting. Members were asked to review each page and provide feedback on design, content and usability. The website was refined based on this additional feedback. The final website was translated into French and reviewed by a French‐speaking member of the Working Group for consistency and usability.

## 3 RESULTS

The Working Group met on five separate occasions in person and virtually (due to the COVID‐19 pandemic) between February 2020 and November 2021. Meetings were 3–5 h in length. Additional collaboration occurred between meetings by e‐mail and smaller group discussions. The composition of the working group is presented in Table 1.

**Table 1:** Working group demographics.

Characteristic	*n* (%)
**Primary role**	
Patient, family and caregiver partner	10 (37.0)
Engagement specialist	4 (14.8)
Researcher	6 (22.2)
Government personnel	7 (25.9)
**Primary organization**	
Ontario Health Team	4 (14.8)
University	6 (22.2)
Government	8 (29.6)
National Health Organization	1 (3.7)
Health System Organization	8 (29.6)

### 3.1 Phase 1: Project scoping and literature review

The Working Group mandate was finalized at the initial working group meeting in February 2020. The group agreed that its activities would focus on building on existing engagement evaluation tools that have emphasized the procedural aspects of engagement,^1^, ^4^ moving towards conceptualizing the outcomes and impacts of engagement and identifying corresponding impact measures. The scope of the work focused on engagement at the planning, service design, governance and policy development levels; engagement at the direct care level was excluded.^18^

The literature review identified several relevant frameworks and tools that were used to collate potential impacts of patient engagement, and existing measures as well as proposed methods for measuring these impacts.^5^, ^6^, ^7^ This information was organized in an Excel database, and grouped into four impact levels: (1) impacts on people, (2) impacts on programmes, (3) impacts on organizations and (4) impacts on health systems. The database was shared with Working Group members for initial prioritizing within categories in advance of engaging with a larger, more diverse set of engagement constituencies across through the concept mapping process.

### 3.2 Phase 2: Concept mapping

**Figure 2:**
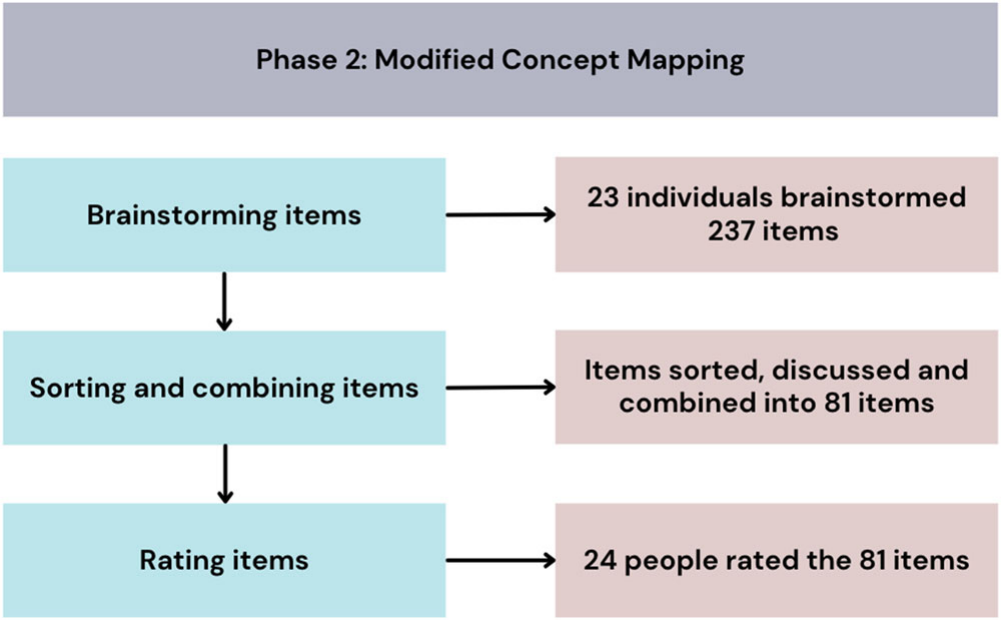
Concept mapping results.

The concept mapping process took place between May and August 2021. The key results of each of the concept mapping process phases are presented in Figure 2. The brainstorming survey was sent to 58 individuals; 23 individuals completed the survey, yielding a response rate of 39.7%. The brainstorming phase yielded 237 impact statements (items), which were sorted and combined by the research team into 81 unique items. The same 58 individuals were invited to rate the importance of measuring each of the 81 unique items from 1 to 10 (1 is least important and 10 is most important) within each of the four levels. Twenty‐four individuals responded to this survey (response rate, 41.4%). The mean importance ratings ranged from 6.1 to 9.7.

### 3.3 Phase 3: Working group deliberations and the measurement framework

Through the Working Group's iterative process of reviewing the literature, seeking feedback from the broader engagement community, and deliberating internally, a final organizing framework was developed that clustered the 81 items around the following conceptual categories (‘domains’) of impact: (1) knowledge and skills; (2) confidence and trust; (3) equity and inclusivity; (4) priorities and decisions; (5) effectiveness and efficiency; (6) patient‐centredness; (7) culture change and (8) patient outcomes and experience. In a separate step, each item was also mapped onto the different impact levels (people, programme, organization and health system) to follow recognized programme evaluation frameworks.

**Figure 3:**
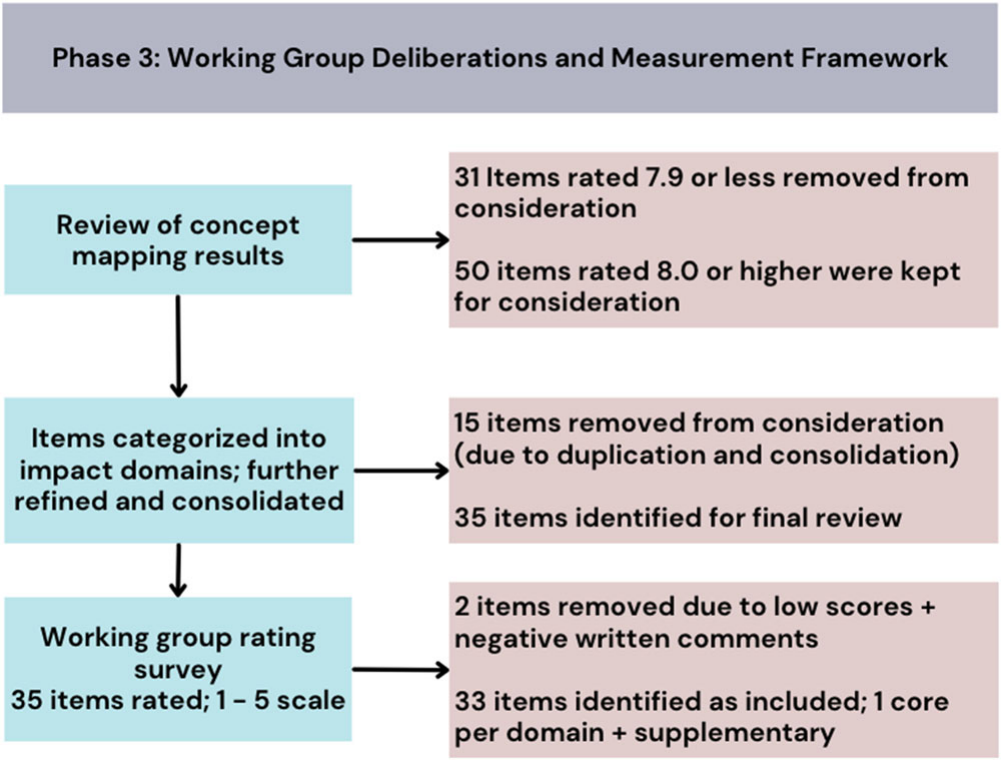
Phase 3 results.

Items that were rated 8.0 or higher out of 10 were identified for inclusion in the ‘go zone’ (i.e., reduced the overall number of items without losing relevant information and fulfilled the face validity criterion) and were included in the next round of discussion. The removal of items rated lower than 8.0 reduced the list to 50 items. A second round of discussion and item consolidation yielded a final list of 35 items for further consideration (Figure 3). A final survey of Working Group members to assess the perceived importance of each of the 35 items across the 8 domains was completed by 15 individuals (average ratings for each item are reported in Table 2). Within each of the domains, the item with the highest importance rating was labelled a ‘core’ item—an item that would be strongly recommended in all evaluations (items in bold in Table 2). Where importance ratings were low and written feedback was negative, items were dropped. For example, the item ʻpatient partners are frustrated by their patient engagement and partnering experiences’ was excluded as the mean importance rating was 3.5 and many respondents took issue with the negative tone of the item, noting comments such as ʻI think we want a set of desirable impacts’. Furthermore, others were concerned about how it could be measured: ʻfrustration may not be relevant in that it might be subjective…the experience of frustration may be skewed due to many variables’. The final set of items includes 8 core items (one per domain) and 25 supplementary items (Table 2). Users of the framework are advised to include all core items in their evaluations as they were identified as the most important in each of the domains. Supplementary items are not ranked but should be included in evaluations based on the impacts that are of interest to the user. Core items related to the equity and inclusivity, priorities and decisions and culture change domains had the highest overall importance ratings (4.7–4.8).

**Table 2:** Mean importance ratings for item statements.

Domain	Item statement	Mean rating (1–5)
Knowledge and skills	**Patient, family and caregiver partners develop new or improve existing skills as a result of their involvement in patient, family and caregiver engagement and partnering activities**	4.1
	Staff gain new knowledge/skills as a result of their involvement in patient, family and caregiver engagement and partnering activities	3.9
	Patient, family and caregiver partners increase their knowledge of the health system as a result of their involvement in patient engagement and partnering activities	3.6
Confidence and trust	**Patient, family and caregiver partners and staff see increased value in the results of the work**	4.5
	When patient, family and caregiver partners are on the team, the work produced has greater credibility among stakeholders	4.2
	Patient, family and caregiver engagement and partnering lead to greater trust among programme providers and participants	4.1
	Staff feel more confident in participating in patient, family and caregiver engagement and partnering activities	3.7
	Patient, family and caregiver engagement and partnering lead to increased trust in the health system	3.5
Equity and inclusivity	**Patient, family and caregiver engagement and partnering contribute to more equitable programmes because they are shaped by a diversity of perspectives**	4.7
	Patient, family and caregiver engagement and partnering contribute to more equitable decision‐making within health systems	4.5
Priorities and decisions	**Patient, family and caregiver engagement and partnering shape the organization's plans/priorities/decisions/policies**	4.8
	Patient, family and caregiver partners influence the final decisions/outcomes	4.8
	Patient, family and caregiver partners are involved in decision‐making	4.8
Effectiveness and efficiency	**Patient engagement and partnering contribute to the design of effective programmes because they reflect the priorities of those who will be using the programmes**	4.5
	Patient, family and caregiver engagement and partnering contribute to cost‐effective programming across the organization	3.3
	Patient, family and caregiver engagement and partnering support the use of healthcare dollars in a fiscally responsible way	3.3
	The organization's patient, family and caregiver engagement and partnering programme is cost‐effective	3.1
Patient‐centredness	**Patient, family and caregiver engagement and partnering make the health system more aware of the patient journey and challenges in the system**	4.4
	Patient, family and caregiver engagement and partnering facilitate the ability for organizations to provide client‐centred services	4.3
	Patient, family and caregiver engagement and partnering contribute to programming that is more patient‐centred	4.3
	Engaging patient, family and caregiver partners increases the relevance of the programme as it reflects the needs of patients/families/caregivers	4.0
	Organizations are better able to offer the right services, in the right way at the right time	3.9
Culture change	**The organization adopts a culture of co‐designing and co‐producing healthcare**	4.8
	Patient, family and caregiver engagement and partnering are sustained beyond the support of an organizational champion	4.5
	Patient, family and caregiver partners are valued by the organization	4.5
	Patient, family and caregiver engagement and partnering become embedded throughout the health system	4.5
	Staff gain a better understanding and appreciation of the issue from the patient, family and caregiver perspective	4.3
	Staff and leadership gain a greater appreciation of the importance of patient, family and caregiver engagement and partnering	4.3
	Patient partners are embedded in all programmes within the organization	4.3
	Patient, family and caregiver engagement and partnering are valued and recognized by the health system	4.3
Patient outcomes and experiences	**Patient, family and caregiver engagement and partnering contribute to improved patient outcomes**	4.5
	Patient, family and caregiver engagement and partnering contribute to improved patient care	4.3
	Patient, family and caregiver engagement and partnering contribute to improved patient satisfaction/experience	4.1

### 3.4 Phase 4: Toolkit web design

**Figure 4:**
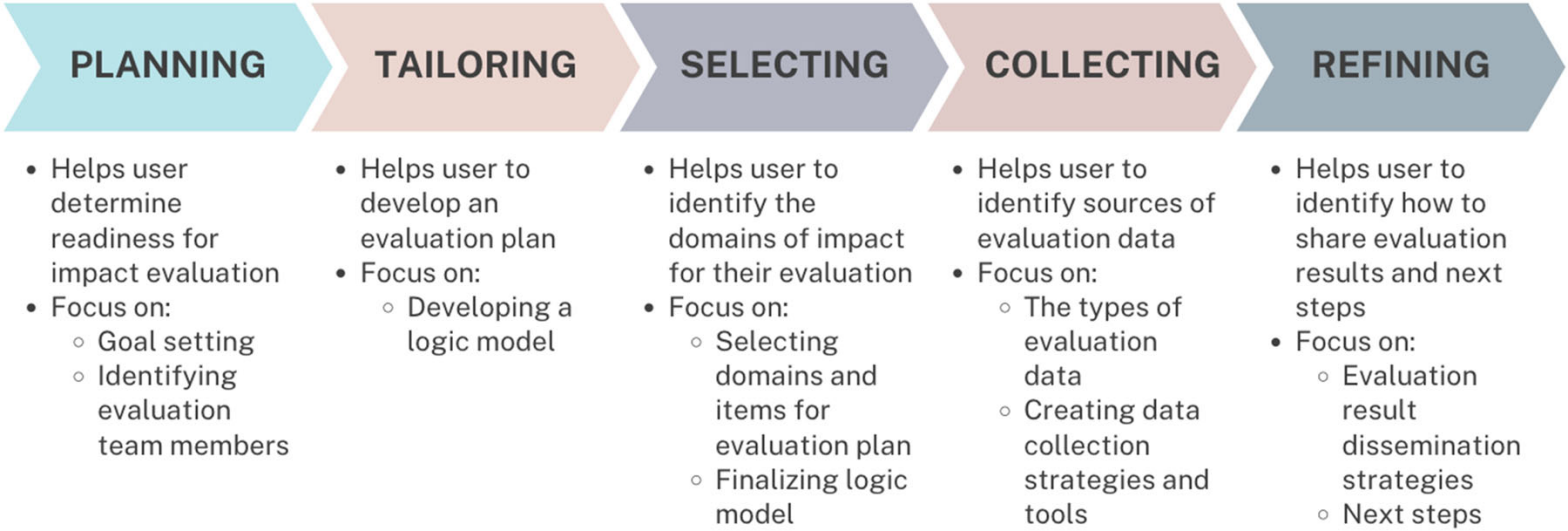
The Engage with Impact Toolkit modules.

The Engage with Impact Toolkit is a module‐based tool that provides users with guidance and support to evaluate patient, family and caregiver engagement, with a focus on impact. The impact framework, including the domains, items and measures, created through the first 3 phases of this work provides the building blocks for the toolkit. The toolkit design was guided by the Working Group principles of ensuring that the product would be practical, accessible and easy to use, menu‐driven and aligned with relevant government and health system priorities. This resulted in a significant expansion in the scope of the work from initially developing an impact framework to developing a more fulsome toolkit on how to support evaluation at each stage of the process (i.e., how to plan for and assess readiness for evaluation, develop an evaluation logic model, select relevant domains and items for evaluation, create a data collection plan and share evaluation results and assess the next steps) (Figure 4).

**Figure 5:**
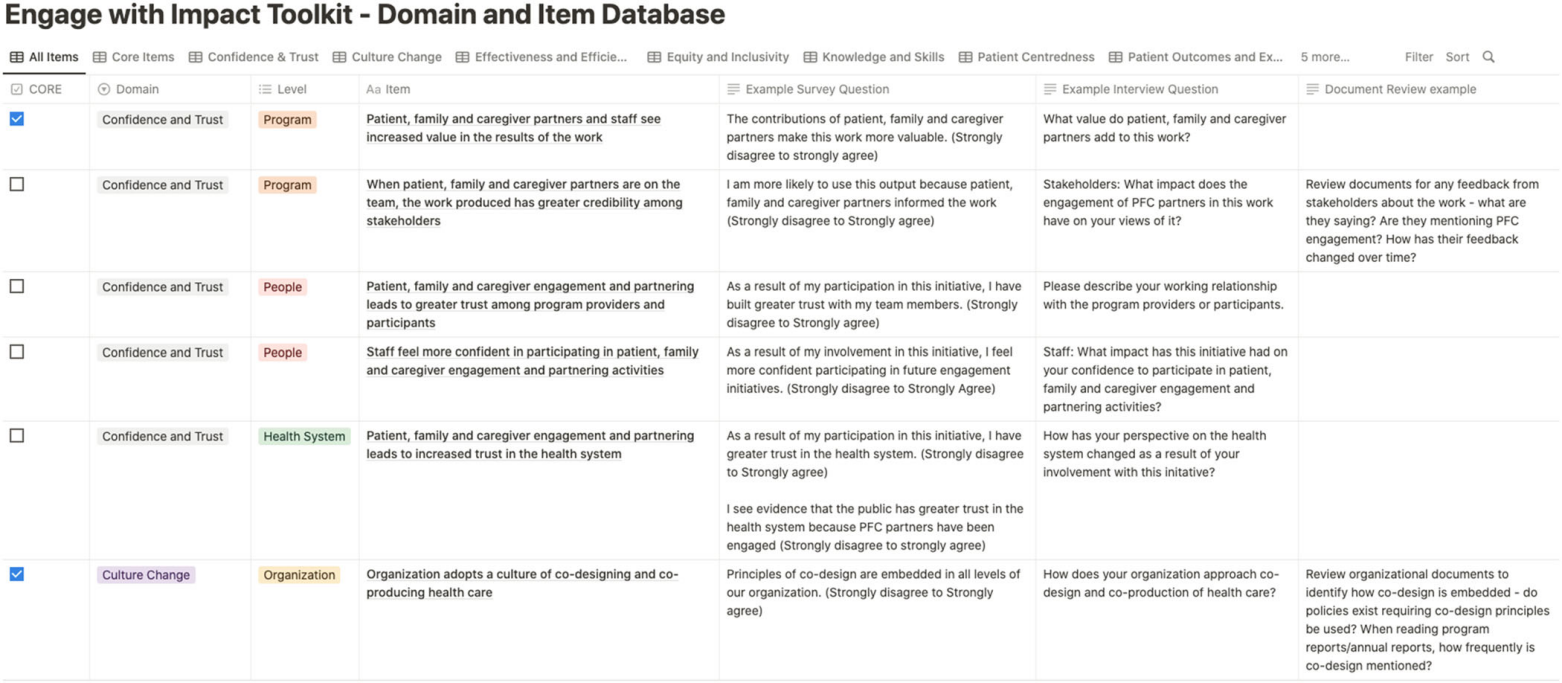
Screenshot of the online database.

The toolkit includes the final set of domains and items generated through concept mapping and working group deliberations (Table 2). Corresponding survey questions, interview questions and/or document review procedures are provided for each item in a separate online database housed within the toolkit (Figure 5). Where available, items and questions from existing evaluation tools identified through the literature search have been used to populate the database, augmented by new or tailored versions as needed. Item wording was also revised in some instances to ensure consistent language use and ease of understanding. The toolkit is a living document and is being continuously revised based on feedback received through the implementation stage, which currently includes requests for feedback on the website itself and, in the future, will include pop‐up surveys embedded on the website and follow‐up interviews with the toolkit users.

The research team developed a prototype website that was shared with Working Group members in October 2021 who provided feedback on its content, design and usability through e‐mail correspondence, one‐on‐one user testing and at the final Working Group meeting. Member feedback focused on ensuring that the content was easy to understand and follow for users at all stages of evaluation; that the website was accessible to all users (e.g., font size, colour selection‐related feedback) and that the website was visually appealing. Additional content suggestions were also made, such as the inclusion of short training videos in each of the modules. Website modifications were made, and the penultimate version was shared back with the Working Group for final comments. The final toolkit content was translated into French by a professional translator. The translated content was reviewed and checked for accuracy and completeness by a French‐speaking member of the working group. Required revisions were implemented.

The full toolkit was launched in January 2022 and can be found at www.evaluateengagement.ca.

## 4 DISCUSSION

As the public and patient engagement field matures, the need for robust but accessible evaluation tools and metrics is a high priority to capture the outcomes and impacts of engagement and to support continuous learning and improvement. Our tool development process builds on current evaluation tools and addresses key deficiencies noted in recent systematic reviews of public and patient engagement evaluation tools, namely, the lack of scientific rigour of tools, the absence of public and patient engagement in tool design,^5^ the benefits of a maturity model approach to evaluation over time, the need for consensus‐building to identify and disseminate core metrics^6^ and the utility of resources that can be adapted and combined in a locally generated co‐design activity.^7^

We reflect on several substantive, methodological and practice‐related aspects of the measurement framework results and the development of the evaluation toolkit as a whole. First, the conceptual mapping of the domains and items in Table 2 offers several insights into the emphasis given to some domains over others. For example, the larger number of items in the *organizational culture* and *trust and confidence* domains reinforces the view of engagement as a journey focused on culture change and a reorientation to patient‐ and family‐centred care, and as relational work that at its core is about relationship‐building and trust.^19^ Second, our final mapping of domains and items reflects the strong desire of our working group members to take a broad lens to measure impacts that include *all* partners (i.e., staff, leadership team members), not just patient, family and caregiver partners. Current approaches to evaluating patient, family and caregiver engagement often focus on evaluating the impacts of patient and caregiver partners specifically, in isolation from other team members. This often places the onus on patient partners to drive change, to be effective partners and to champion engagement, which was viewed as highly burdensome to patient partners and antithetical to an organization‐wide focus on supporting patient engagement. This concern had a strong shaping effect on the final set of domains and impact items, ensuring that there was a balance of items focused on the impacts of patient partners themselves as well as the impacts of staff/organization leads, and on the toolkit design process as a whole.

Methodologically, our adapted concept mapping approach may offer helpful guidance for future co‐design work in the engagement field. In standard applications of concept mapping, all participants contribute to the structured sorting process following the brainstorming stage. Sorting is considered an intensive activity designed to ‘capture participants judgements about the relationship between all items in a typically large set’ and normally, has around a 50% completion rate. Given the unique composition of our Working Group (i.e., mix of patient partners, engagement specialists and researchers) and the challenges that we faced in conducting our meetings during the early stages of the pandemic with varied experiences using online meeting platforms, we chose to streamline the sorting stage. Research team members carried out a preliminary sorting of impacts into domains, which was then shared with the Working Group for confirmation and revision. This adaptation of the method served to reduce participant burden and mediated the attrition that is characteristic of the sorting stage of concept mapping.^20^

At a practice level, we anticipate that the collaborative approach adopted for the development of the measurement framework and toolkit design, with prospective end users involved through all stages, will facilitate its use and uptake across a wide range of health system partners. We are aware of several OHTs, research networks, and other organizations that have begun to use the toolkit to support their engagement work and its evaluation. Early experience with toolkit implementation demonstrates that while users find the website easy to navigate and practical, considerable effort is required to work through the modules and to identify and tailor the metrics to their specific engagement and organizational contexts. This may present barriers for some end users, particularly those with limited evaluation experience and support capacity. Ongoing collaboration with end users, specifically supporting the implementation of the toolkit in various settings, is underway to increase the toolkit's usability including usability surveys, the development of case studies and sample data collection tools.

### 4.1 Limitations

There are several elements related to the execution of this study that may have limited the robustness of the measurement framework. First, the low response rate (40%) obtained for the rating activities is of some concern. Completion rates of between 65% and 70% are more typical for the rating stages of the concept mapping process.^20^ We did not collect information about respondents versus nonrespondents in this activity but offer two possible explanations for the lower response rate. First, rating activities often followed Working Group meetings where preliminary discussions about ratings were held; thus, members may have felt that they had already provided their feedback through meetings and did not want to take the extra step of completing the formal rating survey. A second explanation is that individuals recruited to participate in the rating survey through snowball sampling may have had conflicting priorities, given their organizational roles focused on various pandemic‐related priorities.

A second potential weakness of our study is that participants were asked to rate the items based on their importance and not their feasibility. Including feasibility as one of the rating items is common in concept mapping to keep a focus on actionable items (i.e., highly relevant, easy‐to‐implement items). However, working group members felt that it would be difficult for them to assess feasibility, so it was excluded. The omission of feasibility considerations likely resulted in the identification of items that were challenging to measure. For example, only a very general set of health system‐level impacts was generated, which suggests that this is a priority area of focus requiring further development. Our hope is that by gaining experience using the toolkit's logic model approach, organizations will be better supported to trace the impacts of their engagement work more systematically to be able to assess broader health system impacts. Further, while equity and inclusivity were identified as a priority area for many on the Working Group, a limited number of items were identified in this domain. This may be reflective of the state of the patient and caregiver engagement field, where the considerable focus has been placed on the importance of engaging in an inclusive manner and in pursuit of equity goals, but significant gaps remain in turning principles into action.^21,22^ We anticipate that this section of our toolkit will expand over time as more attention is paid to this area.

## 5 CONCLUSION

As the public and patient engagement field matures, the focus on evaluation is a growing priority to support continuous learning and improvement and to demonstrate the critical links between the involvement of patients, family members and caregivers in system planning and design, and improved patient experience and quality of care. The Engage with Impact Toolkit is an early contribution to the measurement of these impacts. Rigorously co‐developed by patient, family and caregiver partners, engagement specialists and researchers, it is a living resource that will be continuously refined to reflect user experiences and new developments in the field.

## AUTHOR CONTRIBUTIONS

This project was led by Julia Abelson, who was the grant holder and project PI. Julia Abelson, Laura Tripp, Maggie MacNeil and Jeonghwa You contributed to the study design, including the modification of the concept mapping process, concept mapping survey development and implementation and initial data analysis. All other authors contributed to the scoping of the project, the interpretation of the concept mapping data, the development of the impact measurement framework and the core design concept for the Engage with Impact Toolkit website. The website itself was developed by Laura Tripp and Julia Abelson, with input provided by all authors. The first draft of the manuscript was written by Julia Abelson and Laura Tripp. All authors reviewed and contributed to manuscript drafts and approved the final version.

## CONFLICT OF INTEREST STATEMENT

The authors declare no conflict of interest.

## ETHICS STATEMENT

This research was reviewed and approved by the Hamilton Integrated Research Ethics Board, Project #8360.

## Data Availability

The data that support the findings of this study are available from the corresponding author upon reasonable request.
